# Analysis of Correlation between an Accelerometer-Based Algorithm for Detecting Parkinsonian Gait and UPDRS Subscales

**DOI:** 10.3389/fneur.2017.00431

**Published:** 2017-09-01

**Authors:** Alejandro Rodríguez-Molinero, Albert Samà, Carlos Pérez-López, Daniel Rodríguez-Martín, Sheila Alcaine, Berta Mestre, Paola Quispe, Benedetta Giuliani, Gabriel Vainstein, Patrick Browne, Dean Sweeney, Leo R. Quinlan, J. Manuel Moreno Arostegui, Àngels Bayes, Hadas Lewy, Alberto Costa, Roberta Annicchiarico, Timothy Counihan, Gearòid Ò. Laighin, Joan Cabestany

**Affiliations:** ^1^Fundació Privada Sant Antoni Abat, Consorci Sanitari del Garraf, Vilanova i la Geltrú, Spain; ^2^Electrical and Electronic Engineering Department, NUI Galway, Galway, Ireland; ^3^Technical Research Centre for Dependency Care and Autonomous Living (CETpD), Universitat Politcnica de Catalunya, Vilanova i la Geltrú, Spain; ^4^Sense4Care, Parc UPC, Cornellà de Llobregat, Spain; ^5^Unidad de Parkinson y trastornos del movimiento (UParkinson), Centro Médico Teknon, Barcelona, Spain; ^6^IRCCS Fondazione Santa Lucia, Rome, Italy; ^7^Maccabi Healthcare Services, Tel Aviv, Israel; ^8^School of Medicine, NUI Galway, Galway, Ireland; ^9^Holon Institute of Technology, Holon, Israel; ^10^Niccolò Cusano University of Rome, Rome, Italy

**Keywords:** Parkinson’s disease, objective monitoring, accelerometers, gait, UPDRS

## Abstract

**Background:**

Our group earlier developed a small monitoring device, which uses accelerometer measurements to accurately detect motor fluctuations in patients with Parkinson’s (On and Off state) based on an algorithm that characterizes gait through the frequency content of strides. To further validate the algorithm, we studied the correlation of its outputs with the motor section of the Unified Parkinson’s Disease Rating Scale part-III (UPDRS-III).

**Method:**

Seventy-five patients suffering from Parkinson’s disease were asked to walk both in the Off and the On state while wearing the inertial sensor on the waist. Additionally, all patients were administered the motor section of the UPDRS in both motor phases. Tests were conducted at the patient’s home. Convergence between the algorithm and the scale was evaluated by using the Spearman’s correlation coefficient.

**Results:**

Correlation with the UPDRS-III was moderate (rho −0.56; *p* < 0.001). Correlation between the algorithm outputs and the gait item in the UPDRS-III was good (rho −0.73; *p* < 0.001). The factorial analysis of the UPDRS-III has repeatedly shown that several of its items can be clustered under the so-called Factor 1: “axial function, balance, and gait.” The correlation between the algorithm outputs and this factor of the UPDRS-III was −0.67 (*p* < 0.01).

**Conclusion:**

The correlation achieved by the algorithm with the UPDRS-III scale suggests that this algorithm might be a useful tool for monitoring patients with Parkinson’s disease and motor fluctuations.

## Introduction

Although no assessment methods can substitute the clinical judgment, subjective and objective measures in PD complement each other, each method having strengths and weaknesses (1). Objective data from inertial sensors are interesting new way of assessment, with some strengths such as their comparability among physicians, their independence of the observer training, and the fact that their results can be understandable even by the patients (2, 3).

Inertial sensors are of great interest in the case of patients with *motor fluctuations*. These patients experience fluctuations between a state called On, where symptoms are satisfactorily controlled with medication, and a state called Off, where symptoms reappear and patients experience difficulties in motor function (4, 5). As the disease progresses, these motor fluctuations become increasingly frequent and difficult to control with medication, so objective and detailed information about their intensity and chronology could be an invaluable aid for the fine-tuning of the medication.

Accelerometers can detect different motor symptoms and fluctuations in patients with Parkinson’s disease (6–9). Our group earlier developed an algorithm capable of detecting the motor state in patients with motor fluctuations (On and Off) based on accelerometry data from a single inertial sensor located on the patient’s waist. As published before, the algorithm detects whether the patient is walking in Off with specificity and sensitivity of 96 and 94%, respectively, under real conditions of use. To that end, the algorithm first detects gait, then identifies strides and extracts a frequency characteristic of them, which has been shown to be related to the motor state (10). This frequency characteristic consists in the power spectra between 0 and 10 Hz.

Although the motor status has traditionally been classified dichotomously in On and Off states, motor symptoms are a continuum between these two states, and are more precisely scored by numerical scales such as the Unified Parkinson’s Rating Scale part III (UPDRS-III). As the output of the previously developed algorithm is a continuous numerical variable, in this study, we aim to investigate its possible correlation with the UPDRS, to further validate the algorithm.

## Materials and Methods

This prospective study was conducted on a sample of 75 patients suffering from idiopathic Parkinson’s disease, according to the criteria of the UK Brain Bank (11), in moderate stage (Hoehn and Yahr scale >2) with motor fluctuations. Patients older than 80 years and those with implanted electronic devices, dementia, or gait-impairing health problems other than Parkinson’s disease, were excluded from the study. Patients unable to recognize their own On–Off motor states, were also excluded. Participants were selected by convenience sampling among those attending the neurology clinics in any of the participating hospitals: Centro Médico Teknon (Spain), Fondazione Santa Lucia (Italy), Maccabi Healthcare Services (Israel) School of Medicine, NUI Galway (Ireland). We estimated a minimum of 62 patients included to find a significant correlation coefficient of 0.4, considering a <0.05 α error and <0.1 β error. For sample size calculation, the following formula was used (12):

(1) $$N=[(Z_{\text{α}}+Z_{\text{β}}\text{)/C}]\cdot 2+3$$

where *N* is the minimum sample size, the standard normal deviate for α = 0.05 is *Z*_α_ = 1.960, the standard normal deviate for β = 0.1 is *Z*_β_ = 1.282, and *C* = 0.5 × ln[(1 + r)/(1 − r)] = 0.424 being *r* the expected correlation coefficient (0.4).

The study was conducted at the patients’ home and neighborhood. The researchers visited the patients within the time period they typically were in the Off phase (occasionally facilitated by reducing or skipping the previous dopaminergic medication dose). Once the Off state was confirmed by the patient and the researchers, the inertial sensor was placed on the patient’s waist and he/she was asked to walk for some minutes (inside and outside home). More concretely, patients were asked to (I) show their home; if this took less than 2 min, the patient was asked to repeat it; and (II) walk without assistance 10 m. The researchers waited until the patient entered the On phase and repeated the test with sensor. All patients were also administered the UPDRS-III both in Off and On phase. The sensor readings were not available to the researchers at the moment of data collection, and the researchers involved in data collection did not participate in data analysis. We did not consider necessary to blind the UPDRS assessors to the motor’s phase since UPDRS in clinical practice is usually administered by professionals who are aware of the motor phase of the patient at the time of evaluation. The local Ethical Committees approved the research protocol in each study site. All participants signed an informed consent form before their inclusion in the study.

The sensor consisted of a 9 × 2 device, which was worn by patients at the waist through a neoprene belt. This sensor includes a triaxial accelerometer (13). Its measurements were treated by a signal processing algorithm that analyses patient’s gait. The algorithm firstly detects gait by using a machine learning technique (Support Vector Machines), which was trained through labeled signals from 10 PD patients of a previous study (14). Second, the algorithm segments the signals into strides by recognizing specific characteristics on the acceleration measurements. Each individual stride is then characterized through a single frequency feature consisting of the power spectra between 0 and 10 Hz. This feature provides a scalar value whose range is usually 3–6 for patients in Off, and 7–10 for patients in On. In a previous study (10), patients in On state provided higher values of this feature than patients in Off state; hence, this frequency feature is expected to be negatively correlated with UPDRS scores. The algorithm analyses walking bouts with 10 or more strides. Patients during the data collection may walk several times; thus, for each patient and motor state, features obtained in the walking bouts done during a data collection were separately averaged, and the resulting value from each patient and motor state was compared to UPDRS.

For data analysis, bivariate correlations (Spearman) were conducted between the numerical results of the algorithm and the UPDRS-III. More concretely, we obtained the correlation between the algorithm outputs and the total UPDRS-III and, furthermore, between the algorithm outputs and the UPDRS-III items. Every patient was included twice in the same analysis: the first time while he/she was in the Off phase and the second time while in the On phase. For the sake of clarity, we would like to note that each correlation value was obtained by using a single input variable (scalar algorithm results) and a single output variable (UPDRS values, as previously described).

## Results

A total of 75 patients fulfilled the required criteria and their data were complete. The clinical and sociodemographic characteristics of the sample are shown in Table 1.

**Table 1:** Characteristics of the participants.

	Mean	SD
Age	68.6	7.4
Years of disease progression	11.6	1.5

	***n***	%

Women	27	36
Men	48	64
Married	61	81.3
Single/widower	14	18.7
Dyskinesia		
No	28	37.3
Yes	47	62.7

	**Median**	**IQR**

Unified Parkinson’s Disease Rating Scale Part-III*		
Off	40	25
On	15	13
Mini-mental	29	3
H&Y	3	0.5
FOG-Q	13.5	7.5

*p < 0.001.

**Figure 1:**
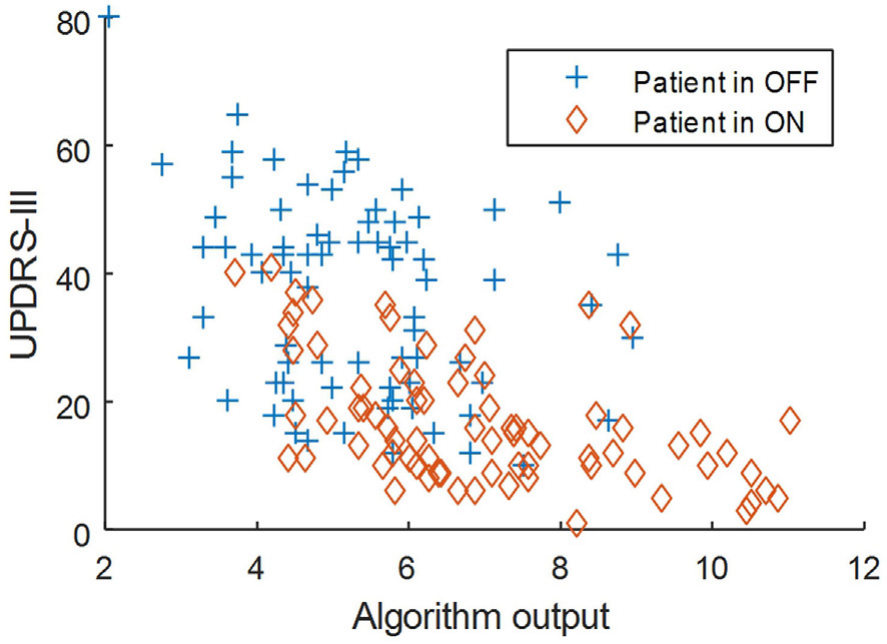
Scatter plot of the algorithm output against UPDRS-III total score.

**Figure 2:**
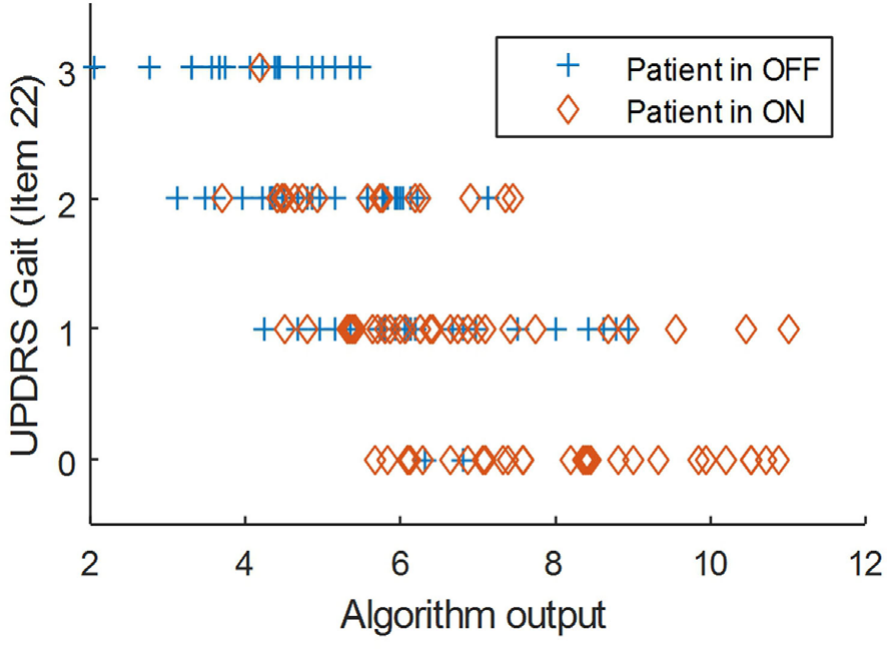
Scatter plot of the algorithm output against UPDRS Gait (item 22).

The correlation between UPDRS-III and algorithm outputs was −0.56 (*p* < 0.001). The correlation with the gait item of UPDRS-III was −0.73 (*p* < 0.001). Figures 1 and 2 show scatter plots of the algorithm output against UPDRS-III total score and against the gait item of the scale. The correlation of the rest of items in the motor section of UPDRS with the algorithm outputs is shown in Table 2. The factorial analysis of the UPDRS-III had previously shown that the following items are clustered in one factor: speech, facial expression, arising from a chair, gait, postural stability, posture, and body bradykinesia [Factor 1: “axial function, balance, and gait” (15)]. The correlation between the algorithm output and Factor I was −0.67 (*p* < 0.01).

**Table 2:** Spearman’s correlation coefficient between the Unified Parkinson’s Disease Rating Scale-motor items and the data from algorithm.

Item #	Description	Rho	*p*
22	Gait	−0.729	**<0.001**
20	Arising from chair	−0.627	**<0.001**
24	Body bradykinesia and hypokinesia	−0.548	**<0.001**
21	Posture	−0.536	**<0.001**
2	Facial expression	−0.469	**<0.001**
1	Speech	−0.464	**<0.001**
10 + 11	Lower extremities rigidity	−0.453	**<0.001**
8 + 9	Upper extremities rigidity (both)	−0.435	**<0.001**
23	Postural stability	−0.419	**<0.001**
18 + 19	Legs agility (both)	−0.340	**<0.001**
7	Axial rigidity	−0.332	**<0.001**
16 + 17	Alternating movements of hands (both)	−0.322	**<0.001**
12 + 13	Finger taps (both)	−0.314	**<0.001**
14 + 15	Hand grips (both)	−0.297	**<0.001**
3	Tremor lower extremities	−0.253	**0.002**
5	Tremor upper extremities	−0.182	**0.026**
4	Tremor face, lips, chin	−0.129	0.116
6	Action tremor of hands	−0.008	0.924

*Bold type: significant results*.

## Discussion

According to the most widely used interpretation of the correlation coefficient, the algorithm outputs are moderately correlated with the UPDRS-III (16). Some items of the UPDRS-III, related to axial function, are well correlated with the algorithm results: gait, arising from chair, global bradykinesia, and posture. Such items are part of the so-called Factor 1 of the UPDRS, which also includes facial expression and postural stability. Therefore, the correlation with the complete Factor 1 of the UPDRS-III is also good. On the other hand, a low correlation is found for items related to tremor and hand grips with values between −0.3 and 0. These low correlations were expected given that the algorithm analyses gait based on acceleration measurements obtained from the waist.

In a similar work, Weiss et al. (17) tested an accelerometer, placed in the lower back, on 22 Parkinson patients while walking. Their sample of patients was about the same age and sex distribution than ours, although in average had less severe disease: H&Y = 2.5; UPDRS motor in Off = 23.6 ± 9.4; and 4.8 (SD3.8) years of disease progression. The test included a 1-min straight walk in On and Off state and a 500-m walk around the hospital at their self-selected speed. This latter test was only performed in On state. They did not find correlations between the acceleration measures and the UPDRS motor or the Hoehn and Yahr scale; however, they found a lower correlation (Pearson’s = 0.5) between the average stride time and a subset of gait related items of the UPDRS (UPDRS-Gait5): falling, freezing of gait, walking, postural stability, and gait. The lack of significant correlation may be due to using the locomotor band of 0.5–3 Hz, which is suitable for acceleration measurements sensed from the lower limbs but does not match the locomotor band from waist measurements (usually quite above 3 Hz, until 10 Hz).

Zwartjes et al. (18) used four accelerometers and gyroscopes in feet, thigh, chest, and arms, to assess bradykinesia in six Parkinson patients. Their methodology is based on analyzing the signals from the four sensors to extract gait parameters, such as step length and step velocity, and other temporal features such as duration of a standing position and some hand movements. They found good correlations between parameters related to step length and step velocity with the item of body-bradykinesia of the UPDRS-III (rho = 0.7); however, they did not investigate global correlations with the UPDRS-III. These results suggest that step length is well correlated with bradykinesia UPDRS; however, the limited number of patients of their study limits the generalization of the results. For this experiment, they used patients with DBS, who were measured under three conditions: “On” (stimulator at the optimal settings), “Intermediate” (stimulator at a stimulation amplitude of 80% of the optimal setting), and “Off” (stimulator Off). Interestingly, the score derived from their algorithms did not differ significantly between the “Off,” “Intermediate,” and “On” states; this makes a difference with our algorithm, whose outcomes identifies the On and Off phases with high validity, as published before (10).

Griffiths et al. (19), using the wrist-worn Parkinson’s Kinetigraph (Global Kinetics Corporation) in 34 patients with Parkinson’s, established the correlation of a *bradykinesia score* with UPDRS-III (Pearson: 0.64); the bradykinesia score was defined as the mean spectral power surrounding the maximum acceleration within a 2-min epoch. For this correlation, a single measure of the UPDRS in On was compared with the average *bradykinesia score* obtained from 10 days of measurement (no data are provided on the severity of the patients’ disease). It does not appear that the UPDRS would have been administered in Off at any time and they do not provide data on patients’ Off time during the 10 days, so it is not clear how much their algorithm correlates with the scale when the patient moves worse. Also, they did not report correlations with specific items or subscales of the UPDRS-III to compare with.

Although other studies with acceleration measurements exist, it is difficult to compare the results, as they did not use correlations with the UPDRS, or they focused on other very specific tasks of the scale, such as tremor or dyskinesia items, which are unrelated to our algorithm.

Classical methods to assess Parkinson’s symptoms include questionnaires that are administered in the office, which collect information reported by the patient, and measurement instruments based on physical exams, such as the UPDRS. The former are affected by memory bias and the latter only record the motor state at the time of exploration (advanced Parkinson’s is a fluctuating pathology; therefore, the symptoms present at the consultation time, may not represent well the whole clinical picture). As a consequence, the classic instrument often used as gold standard is the diary of motor fluctuations, which has to be filled by patients for several days. The problem is that these diaries also have their limitations, as some patients do not recognize their symptoms and patients’ adherence to the method is poor, since recording symptoms’ timeline is a hard task, difficult to complete beyond few days (20). On the other hand, sensors are not subject to memory bias or awareness of symptoms and do not require human intervention so they could be used in the long term if needed. However, sensors-based systems could have usability problems that have to be carefully addressed, and adherence of patient to such systems has to be demonstrated yet.

We agree with those who argue that the correlation of the new objective instruments (sensors), with the classic clinical scales, does not have to be perfect (1). The classical instruments are more qualitative, and are influenced by multiple variables; thus, a high correlation between them and pure quantitative measurements, such as the accelerometer signal, is not expected. In addition, it is the limitations of the classical instruments that prompt researchers to search for new methods of assessment; therefore, the perfect correlation of new methods with the old ones would only indicate that the former are no better than the later. It is to be demonstrated in future studies whether the measurements of the new sensors lead or not to a better clinical control, compared to the traditional methods, although it is likely that, at least in the case of patients who do not recognize their motor fluctuations correctly, sensors would improve the time mapping of the motor phase. Furthermore, we think that the sensors would describe the motor phase better than the diaries, in those moments in which the patients are not clearly in Off or On, but in an intermediate state or switching the phase.

Our algorithm is limited by the fact that it is an algorithm that analyses the patient’s gait, which means that it does not provide data on motor status if the patient does not walk. The authors believe that this limitation is not very important in clinical practice, since patients with Parkinson’s who walk, do so multiple times a day, producing enough data to map symptoms related with axial function (21, 22). In any case, the information of our sensor could be supplemented with additional sensors (for example, in the extremities), in the event that a more exhaustive monitoring of the motor state were required. In addition to this, a further correlation analysis on a more extensive population of PD patients may help to ensure the reliability of the measurements taken from the strides.

The studied algorithm has previously proven to accurately detect the Parkinson’s motor phase (On or Off) (10). Our present results encourage the interpretation that the measurements of the algorithm correspond to the patient’s axial function, especially the influence of bradykinesia on the gait. The observation that the algorithm may monotonically decrease with the UPDRS-III scale suggests that the values offered by the algorithm have a diagnostic value and are more discriminative than the mere dichotomous On/Off classification. Therefore, this kind of algorithm might be an excellent tool for monitoring patients with Parkinson’s disease and motor fluctuations related to patient’s axial function.

## Ethics Statement

The local Ethical Committees approved the research protocol in each study site. All participants signed an informed consent form before their inclusion in the study.

## Author Contributions

AR-M conceived the study, designed the study and drafted the first version of the manuscript AS, CP-L, DR-M, and MMA contributed to the study design, and performed algorithmic work and statistical analysis. They contributed to and approved the final version of the manuscript. SA, BM, PQ, BG, GV, PB, DS and LRQ performed the field work and approved the final version of the manuscript. AB, HL, AC, RA, TC, and GOL contributed to the study design and data collection in their respective study site. They contributed to and approved the final version of the manuscript. JC contributed to the study conception, coordinated the project in the different study sites and approved the final version of the manuscript.

## Conflict of Interest Statement

AR-M, AS, CP-L, JA, and JC are shareholders of Sense4Care, which is a spin-off company, which may commercialize the results of this research device in future. These authors declare that the possible commercialization of the product is a research outcome, not being the design, the analysis, the interpretation of the results, or the conclusions being affected by commercial interests. All other authors declare that the research was conducted in the absence of any commercial or financial relationships that could be construed as a potential conflict of interest.

## References

[1] Bhidayasiri R Martinez-Martin P Clinical assessments in Parkinson’s disease: scales and monitoring. Int Rev Neurobiol (2017) 132:129–82. 10.1016/bs.irn.2017.01.001 28554406

[2] Maetzler W Klucken J Horne M. A clinical view on the development of technology-based tools in managing Parkinson’s disease. Mov Disord (2016) 31(9):1263–71. 10.1002/mds.26673 27273651

[3] Maetzler W Domingos J Srulijes K Ferreira JJ Bloem BR Quantitative wearable sensors for objective assessment of Parkinson’s disease. Mov Disord (2013) 28(12):1628–37. 10.1002/mds.25628 24030855

[4] Fahn S Oakes D Shoulson I Levodopa and the progression of Parkinson’s disease. N Engl J Med (2004) 351:2498–508. 10.1056/NEJMoa033447 15590952

[5] Ahlskog JE Muenter MD. Frequency of levodopa-related dyskinesias and motor fluctuations as estimated from the cumulative literature. Mov Disord (2001) 16:448–58. 10.1002/mds.1090 11391738

[6] Keijsers NL Horstink MW Gielen SC. Ambulatory motor assessment in Parkinson’s disease. Mov Disord (2006) 21(1):34–44. 10.1002/mds.20633 16127718

[7] Patel S Lorincz K Hughes R Huggins N Growdon J Standaert D Monitoring motor fluctuations in patients with Parkinson’s disease using wearable sensors. IEEE Trans Inf Technol Biomed (2009) 13(6):864–73. 10.1109/TITB.2009.2033471 19846382 PMC5432434

[8] Salarian A Russmann H Vingerhoets FJ Dehollain C Blanc Y Burkhard PR Gait assessment in Parkinson’s disease: toward an ambulatory system for long-term monitoring. IEEE Trans Biomed Eng (2004) 51(8):1434–43. 10.1109/TBME.2004.827933 15311830

[9] Hoff JI van der Meer V van Hilten JJ. Accuracy of objective ambulatory accelerometry in detecting motor complications in patients with Parkinson disease. Clin Neuropharmacol (2004) 27(2):53–7. 10.1097/00002826-200403000-00002 15252264

[10] Rodríguez-Molinero A Samà A Pérez-Martínez DA Pérez López C Romagosa J Bayés À Validation of a portable device for mapping motor and gait disturbances in Parkinson’s disease. JMIR Mhealth Uhealth (2015) 3(1):e9. 10.2196/mhealth.3321 25648406 PMC4342689

[11] Hughes AJ Daniel SE Kilford L Lees AJ. Accuracy of clinical diagnosis of idiopathic Parkinson’s disease: a clinico-pathological study of 100 cases. J Neurol Neurosurg Psychiatry (1992) 55(3):181–4. 10.1136/jnnp.55.3.181 1564476 PMC1014720

[12] Hulley SB Cummings SR Browner WS Grady D Newman TB Designing Clinical Research: An Epidemiologic Approach. 4th ed Philadelphia, PA: Lippincott Williams & Wilkins (2013). 79 p. Appendix 6C.

[13] Rodríguez-Martín D Pérez-López C Samà A Cabestany J Català A. A wearable inertial measurement unit for long-term monitoring in the dependency care area. Sensors (2013) 13(10):14079–104. 10.3390/s131014079 24145917 PMC3859110

[14] Samà A Pérez-López C Romagosa J Rodriguez-Martín D Català A Cabestany J Dyskinesia and motor state detection in Parkinson’s disease patients with a single movement sensor. 2012 Annual International Conference of the IEEE in Engineering in Medicine and Biology Society (EMBC) San Diego, CA: IEEE (2012). p. 1194–7. 10.1109/EMBC.2012.6346150 23366111

[15] Stebbins GT Goetz CG Lang AE Cubo E Factor analysis of the motor section of the unified Parkinson’s disease rating scale during the off-state. Mov Disord (1999) 14(4):585–9. 10.1002/1531-8257(199907)14:4<585::AID-MDS1006>3.0.CO;2-3 10435494

[16] Hinkle DE Wiersma W Jurs SG Applied Statistics for the Behavioral Sciences. 5th ed Boston: Houghton Mifflin (2003).

[17] Weiss A Sharifi S Plotnik M van Vugt JP Giladi N Hausdorff JM. Toward automated, at-home assessment of mobility among patients with Parkinson disease, using a body-worn accelerometer. Neurorehabil Neural Repair (2011) 25(9):810–8. 10.1177/1545968311424869 21989633

[18] Zwartjes DG Heida T van Vugt JP Geelen JA Veltink PH. Ambulatory monitoring of activities and motor symptoms in Parkinson’s disease. IEEE Trans Biomed Eng (2010) 57(11):2778–86. 10.1109/TBME.2010.2049573 20460198

[19] Griffiths RI Kotschet K Arfon S Xu ZM Johnson W Drago J Automated assessment of bradykinesia and dyskinesia in Parkinson’s disease. J Parkinsons Dis (2012) 2(1):47–55. 10.3233/JPD-2012-11071 23939408

[20] Papapetropoulos SS. Patient diaries as a clinical endpoint in Parkinson’s disease clinical trials. CNS Neurosci Ther (2012) 18(5):380–7. 10.1111/j.1755-5949.2011.00253.x 22070400 PMC6493659

[21] Rochester L Chastin SF Lord S Baker K Burn DJ. Understanding the impact of deep brain stimulation on ambulatory activity in advanced Parkinson’s disease. J Neurol (2012) 259(6):1081–6. 10.1007/s00415-011-6301-9 22086738

[22] Cavanaugh JT Ellis TD Earhart GM Ford MP Foreman KB Dibble LE. Capturing ambulatory activity decline in Parkinson’s disease. J Neurol Phys Ther (2012) 36(2):51–7. 10.1097/NPT.0b013e318254ba7a 22592060 PMC3934648

