# Corrigendum: Analysis of Correlation between an Accelerometer-Based Algorithm for Detecting Parkinsonian Gait and UPDRS Subscales

**DOI:** 10.3389/fneur.2017.00604

**Published:** 2017-11-14

**Authors:** Alejandro Rodríguez-Molinero, Albert Samà, Carlos Pérez-López, Daniel Rodríguez-Martín, Sheila Alcaine, Berta Mestre, Paola Quispe, Benedetta Giuliani, Gabriel Vainstein, Patrick Browne, Dean Sweeney, Leo R. Quinlan, J. Manuel Moreno Arostegui, Àngels Bayes, Hadas Lewy, Alberto Costa, Roberta Annicchiarico, Timothy Counihan, Gearòid Ò. Laighin, Joan Cabestany

**Affiliations:** ^1^Fundació Privada Sant Antoni Abat, Consorci Sanitari del Garraf, Vilanova i la Geltrú, Spain; ^2^Electrical and Electronic Engineering Department, NUI Galway, Galway, Ireland; ^3^Technical Research Centre for Dependency Care and Autonomous Living (CETpD), Universitat Politcnica de Catalunya, Vilanova i la Geltrú, Spain; ^4^Sense4Care, Parc UPC, Cornellà de Llobregat, Spain; ^5^Unidad de Parkinson y trastornos del movimiento (UParkinson), Centro Médico Teknon, Barcelona, Spain; ^6^IRCCS Fondazione Santa Lucia, Rome, Italy; ^7^Maccabi Healthcare Services, Tel Aviv, Israel; ^8^School of Medicine, NUI Galway, Galway, Ireland; ^9^Holon Institute of Technology, Holon, Israel; ^10^Niccolò Cusano University of Rome, Rome, Italy

**Keywords:** Parkinson’s disease, objective monitoring, accelerometers, gait, UPDRS

**Addition of an Author**

Leo R. Quinlan was not included as an author in the published article. The authors apologize for this error and state that this does not change the scientific conclusions of the article in any way.

The original article have been updated.

## Author Contributions

AR-M conceived the study, designed the study and drafted the first version of the manuscript AS, CP-L, DR-M, and MMA contributed to the study design, and performed algorithmic work and statistical analysis. They contributed to and approved the final version of the manuscript. SA, BM, PQ, BG, GV, PB, DS and LRQ performed the field work and approved the final version of the manuscript. AB, HL, AC, RA, TC, and GOL contributed to the study design and data collection in their respective study site. They contributed to and approved the final version of the manuscript. JC contributed to the study conception, coordinated the project in the different study sites and approved the final version of the manuscript.

## Conflict of Interest Statement

AR-M, AS, CP-L, JA, and JC are shareholders of Sense4Care, which is a spin-off company, which may commercialize the results of this research device in future. These authors declare that the possible commercialization of the product is a research outcome, not being the design, the analysis, the interpretation of the results, or the conclusions being affected by commercial interests. All other authors declare that the research was conducted in the absence of any commercial or financial relationships that could be construed as a potential conflict of interest.

